# Microvasculature on a chip: study of the Endothelial Surface Layer and the flow structure of Red Blood Cells

**DOI:** 10.1038/srep45036

**Published:** 2017-03-24

**Authors:** Daria Tsvirkun, Alexei Grichine, Alain Duperray, Chaouqi Misbah, Lionel Bureau

**Affiliations:** 1 Univ. Grenoble Alpes, LIPHY, F-38000 Grenoble, France; 2 CNRS, LIPHY, F-38000 Grenoble, France; 3 Research Center for Obstetrics, Gynecology and Perinatology. 4, Oparin street, Moscow, 117997 Russian Federation; 4 Univ. Grenoble Alpes, IAB, F-38000 Grenoble, France; 5 INSERM, IAB, F-38000 Grenoble, France

## Abstract

Microvasculatures-on-a-chip, *i.e. in vitro* models that mimic important features of microvessel networks, have gained increasing interest in recent years. Such devices have allowed investigating pathophysiological situations involving abnormal biophysical interactions between blood cells and vessel walls. Still, a central question remains regarding the presence, in such biomimetic systems, of the endothelial glycocalyx. The latter is a glycosaminoglycans-rich surface layer exposed to blood flow, which plays a crucial role in regulating the interactions between circulating cells and the endothelium. Here, we use confocal microscopy to characterize the layer expressed by endothelial cells cultured in microfluidic channels. We show that, under our culture conditions, endothelial cells form a confluent layer on all the walls of the circuit and display a glycocalyx that fully lines the lumen of the microchannels. Moreover, the thickness of this surface layer is found to be on the order of 600 nm, which compares well with measurements performed *ex* or *in vivo* on microcapillaries. Furthermore, we investigate how the presence of endothelial cells in the microchannels affects their hydrodynamic resistance and the near-wall motion of red blood cells. Our study thus provides an important insight into the physiological relevance of *in vitro* microvasculatures.

Interactions between circulating blood components and vessel walls are central to the immune^1^,^2^ and inflammatory^3^,^4^ response, and to processes such as angiogenesis^5^,^6^ or hemostasis^7^. These interactions result from a complex and highly regulated interplay between specific biomolecular adhesion mechanisms at cell/wall interfaces^1^,^3^,^8^, chemoattractant expression^2^,^9^, mechanical properties of the cells^10^,^11^, and fluid stresses arising from hemodynamics^10^,^11^,^12^,^13^. Anomalous interactions between blood cells and the endothelium, i.e. the cellular layer lining the internal lumen of blood vessels, are known to be associated with a number of blood and vascular disorders such as thrombosis, atherosclerosis, diabetes mellitus, or sickle cell anemia^3^,^14^.

*In vitro* studies have proven to be extremely useful in order to unravel the respective roles of mechanical, biochemical and biophysical factors that govern some vascular pathologies^15^,^16^,^17^,^18^. These studies typically rely on microfluidic tools to create networks of channels that recapitulate the microvasculature properties. Such *in vitro* investigations present several important advantages for the rational studies of blood dynamics, cell trafficking and microvascular functions: (i) they solve technical and ethical issues encountered when working on animal models^19^, (ii) microchannels are made from transparent materials and facilitate the use of advanced and high-resolution microscopy techniques, and (iii) experiments are performed under tightly controlled fluid composition and flow conditions. However, these advantages often come at the cost of a partial loss of physiological relevance, in particular regarding cell/wall interactions. To overcome this limitation, several works have proposed designs of *in vitro* microvasculatures that mimic not only the architecture, but also the surface properties of blood microvessels: endothelial cells have been cultured in microcircuits, made of silicone elastomer or hydrogels, in order to form a confluent monolayer on their walls, thus providing perfusable channels bearing a model endothelium, while displaying two- or three-dimensional network architectures^20^,^21^,^22^,^23^,^24^,^25^,^26^,^27^,^28^,^29^,^30^,^31^. Such *in vitro* microvasculatures have been successfully used for the study of *e.g.* angiogenesis^24^,^29^, thrombosis^32^,^33^, platelet^34^ and leukocyte^35^ adhesion, or leukocyte margination^36^.

The functionality of the endothelium formed *in vitro* inside microchannels has been investigated to some extent: endothelial cells have been shown (i) to line the entire lumen of the channels^21^,^23^,^27^,^29^, (ii) to properly express VE-cadherins and form tight cell/cell junctions^27^,^29^, which ensures good barrier functions to the endothelium^21^, and (iii) to display typical surface adhesion molecules such as ICAM-1^22^ and CD31^20^,^21^, as well as Von Willebrand factor (vWF)^20^,^34^, all involved in adhesion and recruitment of circulating cells to the vessel walls. While this demonstrates the excellent level of mimicry of such endothelialized micronetworks, it is however worthwhile noticing that, to the best of our knowledge, no report has been published concerning the presence of the Endothelial Surface Layer (ESL). The ESL is a biomacromolecular layer essentially composed of glycosaminoglycans such as heparan sulfate and hyaluronan, bound to the apical membrane of endothelial cells^37^,^38^. It is now recognized to play a pivotal role in endothelial barrier functions^39^, in chemoattractant binding and presentation^40^, as well as in mechanical sensing of fluid shear stress by endothelial cells^37^,^41^. Being the innermost part of the vessels, the ESL is directly exposed to blood flow and actually constitutes the primary gatekeeper interacting with circulating blood cells. Its thickness, typically reported to be ~500 nm *in vivo*^37^,^40^, is such that it has a quantitative impact on the hydrodynamic resistance of the smallest vessels^42^,^43^, and that it may shield the adhesion molecules present at the membrane level of the endothelial cells^39^,^40^. The latter has been recently pointed out as an overlooked key factor in the initial stages of the adhesion cascade associated with leukocytes recruitment^39^,^40^. Moreover, ESL impairments are known to be associated with a number of vascular disorders^44^,^45^.

The physiological relevance of microvasculatures-on-a-chip therefore crucially depends on whether an ESL is properly expressed in such systems. This question is all the more important that no clear consensus has yet been reached regarding the presence, extent and structure of the ESL on endothelial cells cultured *in vitro*. Indeed, over the last decade, some studies have reported that while the ESL observed *in* or *ex vivo* displays a thickness of several hundreds of nm, endothelial cells cultured *in vitro* either lack^46^ or exhibit a surface layer of a few tens of nm only^47^. In contrast, other recent works conclude to the presence of a thick ESL *in vitro*^48^,^49^, and point out several important factors that may strongly affect the ESL extent and spatial organization, such as the preparation technique used if imaging fixed cells^48^, the culture conditions^48^, and the presence or not of shear flow^50^.

In this context, our work focuses on characterizing the ESL in endothelialized microchannels made of poly(dimethylsiloxane) (PDMS). We show for the first time, using confocal fluorescence microscopy on living cells cultured under steady flow, that a surface-bound layer is present and lines uniformly the lumen of the channels. Moreover, the average thickness of the ESL is found in the range 600–700 nm, which is highly consistent with *in vivo* determinations. We further characterize the internal topography of the endothelialized channels, show how this and the ESL thickness determine the hydrodynamic resistance of the circuit, and study how the presence of the endothelium affects the near-wall motion of red blood cells (RBC).

## Results

**Figure 1:**
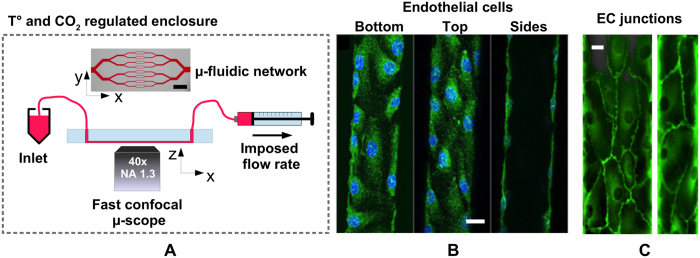
(**A**) Up: low magnification optical image (scale bar: 2 mm) showing the branching/converging architecture of our circuits. For *in situ* live cell imaging, such circuits are constantly perfused at controlled flow rate with culture medium or suspensions of red blood cells, and placed on the stage of a fast confocal microscope equipped with temperature and CO_2_ regulation. (**B**) Confocal images of endothelial cells adhered at the bottom, top and side walls of a microchannel (cytoplasm in green, nuclei in blue). Scale bar: 15 *μ*m. (**C**) Confocal images of VE-Cadherin at cell-cell junctions, at the bottom wall of a channel of 80 × 40 *μ*m (left) and 40 × 40 *μ*m (right) lateral dimensions. Scale bar: 12 *μ*m.

Figure 1A shows the design, adapted from the work of Tsai *et al*.^27^, of the microchannel networks used in this study. They are symmetric and composed of four successive branching (resp. merging) points at which the width of the channels is divided (resp. multiplied) by two, with the middle part of the circuit comprising 16 parallel microchannels of 1 mm length. All channels on a given chip have the same height, and the network geometry is such that the 16 central channels have a square section. We have used microchannels of nominal height 40 *μ*m (for basic cell characterization) and 30 *μ*m (for ESL characterization and flow experiments) in the present study. Circuits were made from a PDMS upper part, fabricated using standard techniques (see Methods), sealed with a glass coverslip at the bottom. The inner surfaces of the channels were then coated with fibronectin, and Human Umbilical Vein Endothelial Cells (HUVEC) were subsequently cultured within the networks, in the presence of a steady flow of culture medium, ensuring a physiologically relevant level of fluid shear stress at the wall of ~0.2 Pa (see Methods).

Confocal Fluorescence Microscopy (CFM) was used to characterize the endothelial layer thus formed (Fig. 1A): as reported in previous works^27^,^30^, HUVEC are seen to adhere on the four walls of the microchannels (Fig. 1B), to be present all along and in every channels of the network, and to form a confluent monolayer with tight cell/cell junctions, as revealed by VE-Cadherin staining (Fig. 1C).

### Endothelial Surface Layer and lumen topography

We have used Wheat Germ Agglutinin (WGA) conjugated with a fluorophore (Alexa 488) to study by CFM the endothelial surface layer expressed by the HUVEC confined inside the microchannels. WGA is a lectin that has been shown to bind to the N-acetyl-D-glucosamine and sialic acid residues of the endothelial surface layer components^51^, and has been used in previous *in vivo*^52^,^53^ and *in vitro*^49^ studies to reveal the ESL. In order to avoid possible artifacts arising from fixation^48^, staining with WGA-Alexa was performed on living cells, and the endothelialized network was subsequently placed on the stage of a confocal microscope, equipped with an environmental enclosure, for 3D imaging.

**Figure 2:**
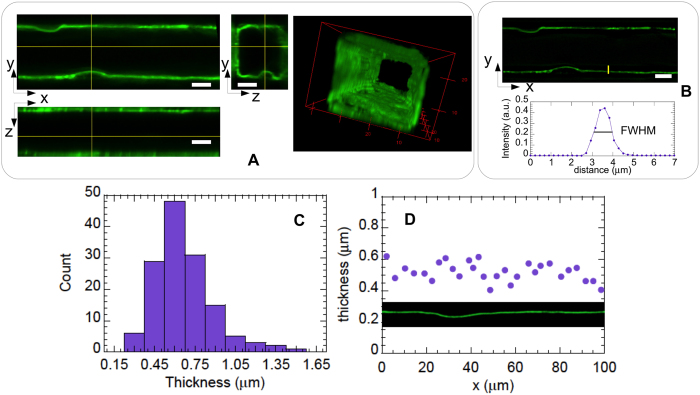
(**A**) Confocal images of the Endothelial Surface Layer (ESL) stained with Alexa488-conjugated wheat germ agglutinin, with views in a horizontal (XY, upper left), and two vertical planes (XZ, lower left; YZ, middle panel), along with a 3D rendering of the imaged channel (right panel). Scale bar is 10 *μ*m. (**B**) XY view of the ESL after image deconvolution by the microscope point spread function (upper panel), and intensity profile, measured along the yellow line drawn on the upper panel, from which the ESL thickness is measured as the full width at half maximum. (**C**) Histogram of the ESL thickness, yielding a thickness of 670 ± 200 nm over 140 measurements. (**D**) ESL thickness measured at 29 different locations along the 100 *μ*m-long image section shown in inset, illustrating the spatial homogeneity of the ESL.

Figure 2A shows that fluorescence is clearly detected at the endothelium surface, and that a fairly thick and homogeneous surface layer, which appears to follow the apical membrane of the cells, entirely lines the lumen of the microchannels. Control experiments further show that HUVEC treated with neuraminidase, an enzyme known to degrade the ESL^49^, exhibit a much dimmer and more heterogeneous surface layer (see Supplementary Information Fig. S1), thus confirming that WGA-Alexa indeed binds to the ESL in our experiments.

We have analyzed images obtained in horizontal (XY) planes in order to determine the thickness of the ESL in the endothelialized microchannels. To avoid overestimating the extent of the ESL, raw images were first deconvolved by the microscope Point Spread Function (PSF, see Methods), after which intensity profiles across the fluorescent layer were plotted and their full-width-at-half-maximum (FWHM) determined and used as a measure of the ESL thickness (Fig. 2B). Measurements were performed over a total of 140 intensity profiles collected in 5 different channels of 30 × 30 *μ*m^2^ in nominal cross section, yielding a thickness of *h*_ESL_ = 670 ± 200 nm (average ± 1 standard deviation), a value much larger than the lateral resolution of the microscope used here. The thickness histogram in Fig. 2C shows that the surface layer could reach up to 1 *μ*m or more in some regions. In spite of variations in thickness from place to place, we have found that a continuous layer covers the endothelial cells, and we did not observe any systematic trend in thickness distribution and spatial organization. As shown in Fig. 2D, the ESL could display a rather uniform thickness, irrespective of the measurement being made atop, around or away from a cell nucleus, and showed no sign of particular spatial structure.

**Figure 3:**
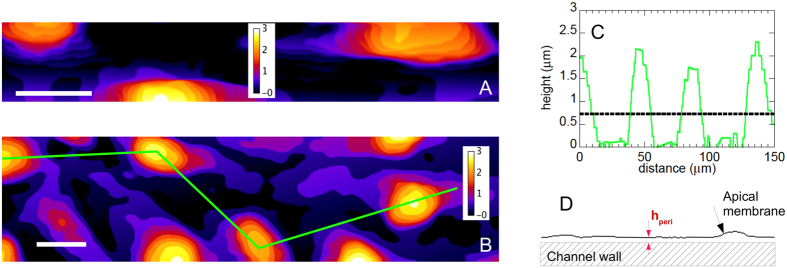
Examples of height maps computed for (**A**) a side wall in a 30 × 30 *μ*m channel, and (**B**) the bottom wall of a 60 × 30 *μ*m channel. Scale bar : 15 *μ*m. Height is color-coded as indicated by the scale in inset (unit: *μ*m). (**C**) Height profile along the green line in (**B**). The horizontal dotted line indicates the value of the profile average height. (**D**) Sketch of the apical membrane of the endothelial cells, located at a minimum distance *h*_*peri*_ of the channel wall.

In contrast to endothelialized microchannels fabricated in a matrix of soft hydrogel^29^, HUVEC cultured in stiffer PDMS channels^27^,^31^ cannot deform or remodel the surrounding material, and cell nuclei induce a roughness at the luminal surface of the channels. As mentioned above and illustrated in Fig. 2, the ESL follows such a surface topography, which we have characterized by processing XYZ image stacks, as described in the Methods section, in order to produce 2D height maps of the HUVEC surface. Examples of such maps are given in Fig. 3. It can be seen that cellular bodies induce small-slope humps that are *h* = 2.3 ± 0.5 *μ*m in height and *w* = 15 ± 5 *μ*m in width (Fig. 3C), with an average spacing of *d* = 35 ± 12 *μ*m (*h, w* and *d* values correspond to mean ± standard deviation over 20 measurements). A topographic analysis of the height maps yields a value of *R*_*q*_ = 0.6 ± 0.1 *μ*m for the root-mean-square (rms) surface roughness and a mean height of *h*_mean_ = 0.7 ± 0.15 *μ*m (*R*_*q*_ and *h*_*mean*_ values correspond to average ± 1 standard deviation computed over 7 different maps). The latter value for *h*_mean_ is given with respect to the lowest points of the profiles, whose height has been set to zero in our topography analysis (see Fig. 3C). The latter point implies that we do not take into account the ESL thickness into *h*_*mean*_, which thus corresponds to the mean height of the HUVEC membrane below the ESL. Moreover, it is important to realize that the points of lowest height of the HUVEC membrane, located at the periphery of the cells far away from the nuclei, actually lie at a finite distance from the channel wall because of the finite thickness of the peripheral cellular regions. In order to determine this distance (see sketch in Fig. 3D), we have analyzed CFM images of HUVEC whose cytoplasm has been stained (see *e.g.* Fig. 1B and Supplementary Information Fig. S2). Following a procedure similar to the one described above for the ESL, we measure a thickness of the peripheral regions of the cells of *h*_peri_ = 0.9 ± 0.2 *μ*m (average ± 1 standard deviation over 66 measurements), in good quantitative agreement with a previous report^54^. Therefore, the actual average distance between the HUVEC apical membrane and the channel wall is 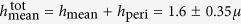
m.

### Red Blood Cell velocimetry

**Figure 4:**
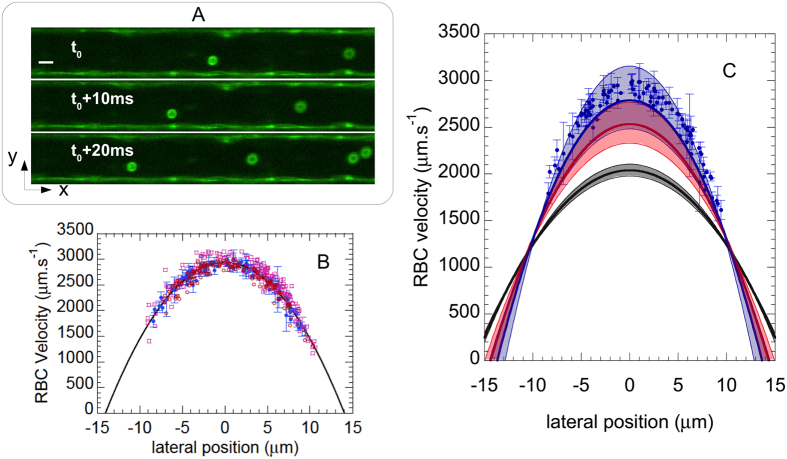
(**A**) Confocal images of Red Blood Cells (RBC) in a channel. Scale bar: 10 *μ*m. RBC positions are shown at a time interval of 10 ms. Flow is from right to left. (**B**) Velocity profiles computed in three different microchannels (blue dots, red circles and purple squares). Error bars plotted for the dataset corresponding to the blue dots represent ± one standard deviation of the velocity measurement on individual RBC trajectories. The black line is a parabolic fit to the latter dataset. (**C**) Experimentally measured velocity profile (symbols), compared to the expected Poiseuille profile, *V(y*) = *V*_max_(1−(*y*/*y*_*wall*_)^2^) for: (i) a bare channel of 32 × 32 *μ*m^2^ cross-section (black line); (ii) a channel of lateral dimension reduced to [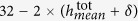] *μ*m (red line), thus accounting for the average thickness of the cell layer (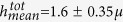
m) and for its roughness (*δ* = 0.065 ± 0.04 *μ*m); and (iii) a channel size of [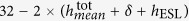] *μ*m (blue line), accounting also for the thickness of the endothelial surface layer (*h*_*ESL*_ = 670 ± 200 nm). The black, red and blue-shaded regions around the solid lines are drawn between the lower and upper estimates of *V(y*) corresponding to the uncertainty on channel dimension and thicknesses measurements.

We have further probed the flow dynamics within our endothelialized channels. A dilute suspension of Red Blood Cells (RBC, ~2.5% in volume fraction) in culture medium was perfused into the channel network at a flow rate of 1 *μ*L.min^−1^, and CFM was used to acquire time-lapse image sequences of the flowing suspension at Z position corresponding to the mid-height plane of the channels. Doing so, we were able to detect simultaneously, using the same excitation and detection settings, fluorescence from the ESL, which indicates the innermost position of the lateral walls, as well as the autofluorescence of the RBC, as illustrated in Fig. 4A. Such image sequences, acquired at a rate of about 300 confocal 2D frames per second, were then processed and analyzed (see Methods) in order to compute the trajectory and velocity (*V*_*RBC*_) of individual RBC flowing at various lateral positions (*y*) in the channels. This allowed us to build *V*_*RBC*_(*y*) flow profiles, as illustrated in Fig. 4B. It can be seen on this figure that *V*_*RBC*_(*y*) profiles, measured in several channels of 30 × 30 *μ*m^2^ nominal section, are in quantitative agreement from channel to channel and display the typical parabolic shape expected for Poiseuille flows. This indicates that, at the low suspension concentration used here, RBC can be used as flow tracers that do not significantly perturb the shape of the expected profile for newtonian fluid flow, as previously shown^55^.

Under the imposed flow rate of *Q* = 1 *μ*L.min^−1^, we measure a maximum flow velocity of *V*_max_ = 2950 ± 200 *μ*m.s^−1^ at the center of the channels (average ± 1 standard deviation over 1100 velocity measurements made at ± 1 *μ*m about the axis in 3 different channels). We now compare such a measured *V*_max_ with theoretical expectations as follows. As mentioned above, our micronetwork comprises, in its central part where velocity measurements have been performed, 16 channels of cross-section *S* in parallel. The flow rate in each of these channels is thus *Q*/16, the average velocity is given by *V*_*avg*_ = *Q*/(16*S*), hence a theoretical maximum velocity:


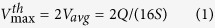


If we compute such a velocity using the actual cross-section of the bare channels, which we measure to be *S* = (32 ± 0.5 *μ*m)^2^ (see Supplementary Information Fig. S5), we obtain 
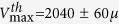
m.s^−1^ (uncertainty on 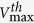 corresponds, here and in the following, to the uncertainty on channel actual dimensions). The measured flow velocity is therefore significantly larger than theoretically expected when taking for *S* the section of the bare channels (Fig. 4C). Besides, control experiments performed in non-endothelialized channels showed that velocity measurements were in excellent agreement with 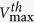 computed as above (see Supplementary Information Fig. S6). We therefore attribute the difference in measured and expected *V*_max_ to the presence of the HUVEC inside the channels, which affects the flow dynamics.

As shown in the previous section, the presence of the cells decreases the size of the available lumen and induces surface waviness. In the present experiments, the Reynolds number is low (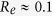), and the roughness of the endothelium is of low amplitude (see previous section, 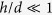). Under such conditions, for which no detachment of the streamlines from the boundary is expected^56^, it has been shown theoretically, for a laminar flow, that the effect of a small-slope surface roughness can be described as a shift of the no-slip boundary condition towards the flow region^57^. This shift is of magnitude 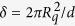, with *R*_*q*_ the rms surface roughness and *d* a typical roughness wavelength^57^. Overall, the no-slip boundary condition is thus predicted to be located at a distance *h*_*mean*_ + *δ* from the valleys of the surface profile.

We use such a prediction in order to account for the wall topography and compute the theoretical maximum velocity in a channel whose lateral dimension is 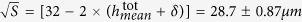, with 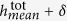 determined from the topography analysis presented above. Doing so, we obtain a value of 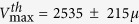
m.s^−1^. While this lies closer to the measured maximum velocity, it still underestimates the experimental results (Fig. 4C). If we account also for the thickness of the ESL in our estimate, by reducing the channel lateral dimension down to 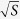 = [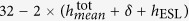] = 27.4 ± 1.07 *μ*m, we compute a theoretical maximum velocity 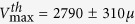
m.s^−1^, which now agrees well, within uncertainties, with the measured value of 2950 ± 200 *μ*m.s^−1^ (Fig. 4C).

### Flow structure

**Figure 5:**
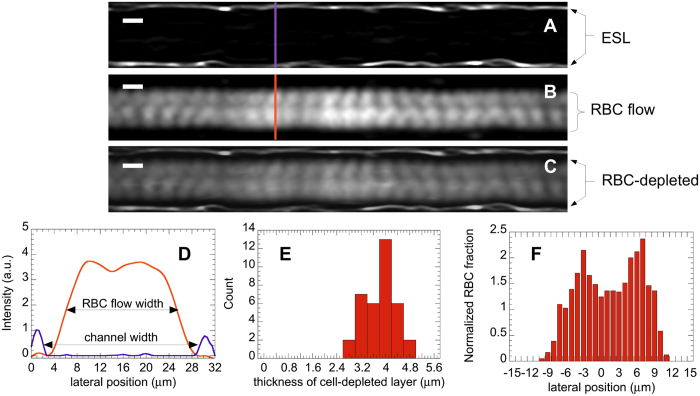
T-projection of a stack average intensity (**A**), standard deviation of intensity (**B**), and overlay of the two (**C**) showing the Endothelial Surface Layer (ESL) at the walls, the flow paths of Red Blood Cells (RBC) in the center, and the dark, RBC-depleted zone near the walls. Scale bar: 10 *μ*m. (**D**) Intensity profiles along the purple and orange lines drawn on (**A**) and (**B**). The channel width is measured as the distance at mid-height between the two lateral ESL peaks, and the RBC flow width is taken as the width at mid-height of the broad central bump. (**E**) Histogram of the measured thickness of the RBC-depleted layer, yielding a thickness of 3.8 ± 0.5 *μ*m over 36 measurements. (**F**) Lateral distribution of RBC fraction (computed over 1200 flowing cells) showing a double peak with a minimum near the channel center.

We have used the acquired time-lapse image sequences (which we call XYT stacks), analyzed above for flow profile computations, in order to investigate the structure of the RBC flow in the endothelialized microchannels. Image stacks, acquired at mid-height of the microchannels, have been processed (see Supplementary Information Fig. S3) by performing two types of intensity projections along the time coordinate (T-projections), in order to obtain, from a given XYT stack, two images containing different information. A T-projection of the stack average intensity produces an image that leaves visible only the stained ESL at the channel wall (Fig. 5A). In contrast, a T-projection of the standard deviation of the intensity is sensitive only to fluctuations caused by the flowing RBC and yields an image that reveals the RBC paths accumulated over the duration of the sequence (Fig. 5B). An overlay of the two images obtained after average and standard deviation projections, as provided in Fig. 5C, thus allows us to superimpose the position of the ESL and the RBC paths.

First, it can be seen on such an overlay that, near the ESL, there is a dark region where intensity fluctuations are close to zero. This, as discussed in details in Supplementary Information (Fig. S4), results from the existence of a near-wall layer that is depleted in RBC. In order to quantify the extent of this layer, we have plotted intensity profiles across the channels, as illustrated in Fig. 5D, and determined (see Supplementary Information Fig. S4) the inner width of the channel (*w*_*ch*_), measured from ESL to ESL, along with the width of the flowing RBC column (*w*_*RBC*_), from which we compute the thickness of the depleted layer as *h*_*dep*_ = (*w*_*ch*_ − *w*_*RBC*_)/2. Doing so over twelve different profiles along a channel, and for 3 different channels, we thus obtain a value of *h*_*dep*_ = 3.8 ± 0.5 *μ*m (see Fig. 5E). Adding to this the average thickness of the ESL layer reported above, we get a thickness of 4.5 ± 0.7 *μ*m for this cell-free layer, measured between the average position of the HUVEC apical membrane and the edge of the flowing RBC column.

Second, it can be noticed in Fig. 5B, in the central region of the channel where RBC are flowing, that the magnitude of intensity fluctuations is modulated quasi-periodically, both streamwise and cross-streamwise. Under the present experimental conditions, T-projections of the standard deviation of intensity are computed from about 400 individual cells traveling in the imaged plane, with each flowing cell being visible in 20 to 30 successive frames in the stacks. Taking ~40 *μ*m^2^ as the surface of individual RBC, the traveling cells therefore cover a cumulated area of 3.2 × 10^5^ to 4.8 × 10^5^ *μ*m^2^, which is 45 to 70 times the surface of the imaged channel section (~7000 *μ*m^2^). At such large ratio values, if the positions of the RBC were uniformly distributed along the X and Y coordinates, one would therefore expect no regular pattern to emerge from projections of the intensity or its standard deviation. Here, the observed streamwise oscillations most likely reflect the fact that, at the flow and image acquisition rates used for the experiments, RBC move by a finite distance, on the order of their size, in between two successive frames, thus producing a modulation of period ~10 *μ*m along the flow direction. More interestingly, we also observe a cross-streamwise modulation, which is visible on the intensity profile on Fig. 5D as a double peak with a local minimum close to the central axis of the channel. As mentioned above, this is unlikely to result from a too small number of imaged RBC. Rather, this suggests that the distribution of RBC across the channel, far from the depleted layer, is non-uniform: RBC appear to have two preferred paths that lie on each side of the channel axis. In order to confirm this and ensure that such an apparent flow structure is not resulting from any artifact of our image treatment, we have built a cross-stream RBC distribution as follows. We have split the channel width into 30 adjacent bins of size Δ*y* = 1 *μ*m, and counted the number of RBC trajectories (*i.e.* centers of mass) detected in each bin, which provided us with a number of RBC, *N*_*RBC*_, as a function of the lateral position, *y*, in the channel. The obtained *N*_*RBC*_(*y*) were then converted to a “linear number density” by dividing by the bin size Δ*y*, and normalized by the average number density, defined as the total number of RBC trajectories detected across the channel, *N*_*tot*_, divided by the mean channel width (*i.e.* [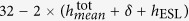] *μ*m, as computed above). Such a normalized distribution, computed over 1200 trajectories and 3 different channels, is presented on Fig. 5F. As suggested above, we find that the RBC distribution displays two off-axis maxima at which the local RBC fraction reaches up to twice the average, with a distance between these two maxima on the order of 8–9 *μ*m.

## Discussion

We have shown here that endothelial cells confined within microchannels and submitted to a physiologically relevant level of fluid shear stress exhibit a glycocalyx that lines the entire lumen of the channels. This provides, to the best of our knowledge, the first evidence for the presence of such a thick and continuous endothelial surface layer in this kind of *in vitro* microvasculature models, showing strong similarities with recent *in vivo* observations in rat and mouse capillaries by Yen *et al*.^58^.

*In vitro* microvasculatures made of endothelialized microchannels have recently emerged as a highly promising tool for in-depth studies of confined blood flows. Such reductionist experimental systems may present specific limitations for investigating the barrier functions of the endothelium: for instance, transport of molecules or migration of flowing cells through the endothelium are hampered when using microchannels made of stiff PDMS, and such studies would require channels formed in tissue-mimicking hydrogels. However, on-chip microvasculatures are perfectly suited for mechanistic studies focusing on interactions between circulating blood components and vessel walls, which are expected to be influenced, among other factors, by the endothelial surface layer.

Our quantification of the thickness of the surface layer relies on the use of confocal fluorescence microscopy performed on living cells whose ESL has been stained, as pioneered by Barker *et al*.^49^. We find an average thickness of the ESL of 670 ± 200 nm, which compares very well with *in* or *ex vivo* measurements of the ESL from hamster cremaster muscle capillaries^59^, mouse cremaster venules^46^, or human umbilical vein^47^, all reported to lie in the range 500–800 nm. A comparison with other *in vitro* studies is less straightforward, due to the large scatter of previous results^48^. Our measurement is in good agreement with a report of 800 nm for human aortic endothelial cells^60^, whereas it lies well above the thickness of 20–30 nm observed by Potter & Damiano^46^ and Chappell *et al*.^47^ on HUVEC, and is found to be quite below the results obtained with confocal microscopy on HUVEC^49^, bovine aortic and rat fat pad endothelial cells^48^, which were reported to lie in the range 1–2.5 *μ*m. While a clear explanation for the low values observed by Potter & Damiano and Chappell *et al*. still needs to be established, Ebong *et al*. have suggested in a recent study that they might result from the specific culture conditions employed, such as the absence of shear flow during culture, or the use of collagen as the protein being bound to the culture substrates prior to cell seeding, which might affect the extent and structure of the ESL when compared to that obtained with cells seeded onto fibronectin-coated surfaces^48^. As far as the agreement of our measurement with other CFM-based studies is concerned, it is important to note that previous works have analyzed cross-sections of the ESL obtained in XZ planes^48^,^49^, for which optical sectioning limits the axial resolution to about 1 *μ*m at best. In contrast to this, we analyze images of the ESL obtained in XY planes, in which the lateral space resolution of a confocal microscope is three to four times better. Moreover, we have taken care here to account for the PSF of the microscope in our image analysis scheme in order to avoid overestimating the ESL thickness, while such a precaution is not mentioned in previous works. Overall, we believe that quantitative differences between our result and previous reports are likely to arise due to both the refined space resolution and image analysis scheme used here, which provide us with an estimate of the ESL that more closely matches *in vivo* measurements.

Building upon the above evidence for a thick ESL in our endothelialized microchannels, we have used them to perform a study of the flow dynamics of red blood cells. Rather than addressing a specific biological question related to the adhesive interactions between circulating cells and the endothelium, as was done successfully in recent works using model microvasculatures^32^,^34^,^35^,^36^, our rationale here was to assess whether we could retrieve, with such biomimetic channels, some of the salient features observed *in vivo* for the flow of RBC confined in microvessels.

In this spirit, we have performed velocimetry experiments using a fast confocal imaging technique, taking advantage of the RBC autofluorescence in order to use them as flow tracers. This has allowed us to build velocity profiles within the endothelialized channels, and to compare them to expected Poiseuille profiles. Such a comparison indicates that experimental flow profiles can be quantitatively described, provided that both the surface roughness and the extent of the ESL are taken into consideration in the effective lumen dimension. This suggests, in good agreement with *in vivo* experiments^61^ and with a recent study performed with glass microcapillaries coated with synthetic polymer brushes^62^, that the ESL does play a role on the hydrodynamic resistance of endothelialized microchannels.

Furthermore, we have shown, using dilute RBC suspensions, that the time-averaged picture of the RBC flow paths can provide valuable information regarding the flow structure. We have thus been able to highlight the existence of a near-wall layer, extending ~4.5 *μ*m away from the apical membrane of the endothelial cells, that is depleted in RBC. Such a layer is reminiscent of the Cell-Free Layer (CFL) that has been observed both *in vivo*^63^,^64^,^65^,^66^ and *in vitro*^55^,^65^,^67^, and has been documented to play an important role in *e.g.* oxygen and nitric oxide transport in arterioles^65^. The formation of the CFL, and its steady-state thickness, is controlled by the balance of (i) the non-inertial hydrodynamic lift force experienced by the deformable RBC flowing close to the vessel walls, which tends to repel them towards the center of the vessel^68^,^69^, and (ii) the cell/cell hydrodynamic interactions, which are at play down to hematocrit as low as 1–2%^68^ and lead to a cross-stream shear-induced diffusion that spreads the RBC distribution across the channel width^68^. Studies of the CFL *in vivo* agree on a thickness of such a depletion layer on the order of 10% of the channel lateral dimension at a physiological hematocrit of 45%^63^,^64^,^67^, and report that the CFL is thicker at lower RBC fractions^63^,^67^ and lower average shear rates^64^. While there is no previous determination of the CFL thickness at the same low hematocrit of 2.5% used in our study, the extent of the CFL measured *in vivo*, in vessels of comparable dimensions (*D*~30 *μ*m in diameter) and under a similar average shear rate (*V*_*mean*_/D~40–50 s^−1^) as those used here, is found to be of 4.7 ± 0.7 *μ*m at the lowest hematocrit of 8% investigated^67^. While the striking quantitative agreement between such a value and that obtained in the present study is likely to be fortuitous, we can however conclude that our determination of the CFL thickness is highly consistent with *in vivo* measurements under close experimental conditions. The latter point is all the more important that, in a careful comparison of *in vivo* and *in vitro* experiments performed in glass capillaries, Suzuki *et al*.^67^ have observed quantitative differences between CFL thickness measurements, which led them to conclude that bare glass microchannels do not provide a reliable *in vitro* model for the study of the CFL. Along the same line, a recent theoretical work focused on numerical simulations of cell migration in small vessels has reported discrepancies between numerical predictions and *in vivo* measurements of the CFL thickness, and tentatively attributed such differences to the fact that the ESL was not accounted for in the simulations^70^. In this context, the agreement between our *in vitro* determination of the CFL thickness and previous *in vivo* results provides a further confirmation of the good level of mimicry of endothelialized microchannels, and exemplifies their interest as realistic microvasculature models.

Finally, our analysis of the cross-stream spatial distribution of flowing RBC has revealed a non-uniform cell distribution exhibiting two main peaks about the channel axis. Under strong enough confinement, *i.e.* when the lateral dimension of the channel corresponds to only a few times the size of the circulating red blood cells, the above-mentioned balance between hydrodynamic lift at the wall and shear-induced diffusion due to cell/cell interactions is expected to lead to a multi-file structure of the flow. This is evidenced in numerical simulations showing that, far from the CFL, the lateral RBC distribution exhibits distinct peaks separated by a distance comparable to the size of the RBC^69^. We are therefore prompted to attribute the flow structure observed in our experiments to such a combination of confinement and hydrodynamic interactions. This is further supported by very recent numerical simulations which show that, under a lateral confinement comparable to that of our experiments and at an average hematocrit as low as 5%, the concentration profile of flowing RBC is predicted to exhibit a minimum at the center of the channel and two lateral peaks separated by a distance on the order of the RBC size^71^. Our observation thus represents one of the very few experimental examples of such a flow structure under confinement.

Overall, the present work complements previous characterizations of endothelialized microchannels by providing evidence for the presence of an endothelial surface layer, which strengthens the physiological relevance of such *in vitro* microvasculature models. Moreover, our study of the flow of healthy red blood cells, which clearly calls for further and more detailed investigations, still illustrates the excellent level of agreement that can be reached between *in vivo* and *in vitro* behaviors when using such biomimetic microfluidic systems. We believe that our results, added to those already obtained in previous works relying on similar devices, set exciting grounds for future studies of blood microcirculation.

## Methods

### Microfluidics

Microchannel networks were fabricated using a standard soft-lithography technique. A master mold of the network was obtained from a positive photoresist (SU8) cast onto a silicon wafer and exposed to UV light through a quartz-chromium photomask. PDMS (Sylgard 184) was cast onto the mold and left for curing for 2 hours at 65 °C. A glass coverslip (#1, thickness 150 *μ*m) was used as the bottom part of the microchip and was permanently sealed to the PDMS upper part after exposure of the surfaces of both elements to an oxygen plasma. The thickness of the photoresist on the master mold was set such that the series of 16 parallel microchannels in the central part of the network had a square section. The actual cross-section of the channels was measured as described in Supplementary Information (Fig. S5). The circuit was connected with silicone tubings to a fluid reservoir at the inlet, and at the outlet to a 1 mL syringe installed on a high precision syringe pump (KDS Legato 110) used in withdraw mode at imposed flow rate.

### Cell culture and staining

Pooled donor Human Umbilical Vein Endothelial Cells (HUVEC) were obtained from Lonza (Switzerland) and cultured in endothelial cells growth medium supplemented by growth factors (EGM^TM^-2 BulletKit^TM^, Lonza, Switzerland). Before cell seeding, microchannels were activated by exposure to an oxygen plasma (Diener Femto, Germany) and coated with human fibronectin (50 *μ*g/ml, PromoCell) for 30 min at 37 °C). Endothelial cells were suspended in culture medium at a concentration of 0.5–1.0 × 10^6^ cells/ml and filtered through a nylon cell strainer with pore diameter of 40 *μ*m. The cell suspension was injected into the microchannel network with a syringe pump with several pulse waves of 30 *μ*L/min alternated with periods of low flow rate of 1 *μ*L/min. Cells were seeded for two consecutive days and subsequently cultured, up to two weeks, in a CO_2_ incubator under a constant flow of supplemented medium at 0.5 *μ*L/min, corresponding to a wall shear stress of about 0.2 Pa (representative of physiological conditions for venous endothelial cells) in channels of nominal section 30 × 30 *μ*m.

For confocal microscopy we used the following dyes: (i) CellTracker Green CMFDA (Molecular Probes^TM^) to label the cytoplasm, (ii) Hoechst 33342 (Molecular Probes^TM^) for nuclei staining, (iii) mouse monoclonal antibody TEA 1.31 to human VE-cadherin was applied as described previously^72^ to label specifically intercellular junctions on fixed endothelium and (iv) Wheat Germ Agglutinin (WGA) Alexa Fluor 488 Conjugate (5 *μ*g/ml, Molecular Probes^TM^) for staining the glycocalyx on living cells. The dyes were used in accordance with manufacturer’s specification. Living cells were stained inside a CO_2_ incubator under flow and appropriate dyes were diluted in preconditioned non-supplemented endothelial cells culture medium to avoid cell retraction and monolayer destruction. Staining (i) was performed on live cells, after which cells were fixed with 4% paraformaldehyde in PBS for 30 min at 37 °C, and then labeled with Hoechst and anti-VE-Cadherin.

For glycocalyx modification, cells were exposed to Neuraminidase from Clostridium perfringens (1U/ml, Sigma-Aldrich) for 60 min in a CO_2_ incubator. Neuraminidase was diluted in non-supplemented endothelial cells culture medium. Glycocalyx was stained by WGA-Alexa 488 before enzymatic modification and fluorescence intensity was estimated before and after Neuraminidase action.

### Blood samples

Whole blood samples were provided by the Etablissement Français du Sang (EFS Rhône-Alpes) from healthy donors. RBC were separated by centrifugation and washed twice in phosphate buffer saline (PBS), after which they were suspended in non-supplemented HUVEC culture medium at low volume fraction for microfluidic experiments. The actual hematocrit (the RBC volume fraction) was estimated directly within the microchannels as follows. We acquired time-lapse sequences, of equal duration Δ*T*, of RBC flowing in 7 XY planes separated by 5 *μ*m in the Z direction, thus spanning the full height of a channel. We counted the number of flowing cells detected in all the imaged planes to obtain the total number of RBC, *N*, traveling through a given channel during Δ*T*. The hematocrit in the imaged channel was then computed as *Ht*(%) = *Nv*/(*Q*Δ*T*) × 100, with *Q* the imposed volumetric flow rate in the channel, and where *v* = 90 fL was taken as the average volume of individual RBC. Doing so, we measured *Ht* = 2.5 ± 0.5% over the three microchannels in which RBC flow was investigated, with *N* = 850 ± 170 RBC counted in each channel during Δ*T* = 3s.

### Confocal imaging

Confocal fluorescence microscopy was performed on an inverted microscope equipped with temperature and CO_2_ control for imaging of live biological objects.

XYZ image stacks of the ESL were obtained in raster mode using a Zeiss LSM710 module and a 40x/NA1.3 oil-immersion objective, with a lateral size of 512 × 512 pixels, a lateral resolution of 0.225 *μ*m/pixel, and a Z-slice spacing between 0.5 and 1 *μ*m.

XYT time-lapses were acquired in fast linescan mode with a LSM7 LIVE module, using a 40x/NA1.0 water-immersion objective, a lateral image size of 512 × 100 pixels and a resolution of 0.444 *μ*m/pixel, at rates in the range 275–360 frames per second.

### Image analysis and processing

The acquired image stacks were processed and analyzed using the Fiji open-source platform and its built-in plugins^73^.

3-dimensional XYZ image stacks of the ESL were treated as follows. A point spread function (PSF) of the microscope was generated numerically with Fiji, using the “Diffraction PSF 3D” plugin fed with the experimental characteristics of our imaging system (*i.e.* refraction index of immersion liquid, objective numerical aperture, lateral magnification and *z* slice spacing of the stack). We have further checked for the consistency of such a generated PSF by using it to deconvolve images of fluorescent latex beads of known diameter (500 nm) and ensuring that the bead size measured from the deconvolved images agreed with the expected one. PSF deconvolution was then performed on image stacks of the ESL using the “Iterative deconvolve 3D” plugin, after which ESL thickness measurements were performed as described in the text.

For topography analysis, the above stacks were processed with an edge-detection filter, thresholded and skeletonized in order to obtain a stack of binary images of the HUVEC apical membrane position, which was then imported as a height map with the “XYZ2DEM importer” plugin and analyzed with “SurfcharJ” package for roughness computation.

Time-lapse XYT image stacks for flow velocimetry were processed as described in detail in Supplementary Information. Raw images were first filtered with a gaussian-blur kernel of radius equal to 3 pixels, yielding images where the moving cells displayed an intensity maximum at their center (see Supplementary Information Fig. S3). The filtered stacks were then analyzed with the “TrackMate” plugin, using the DoG (differences of gaussian) particle detector and the “simple LAP tracker” for building trajectories. The output of this analysis was, for each detected particle, the trajectory, its average *y* position in the image, the average speed along the trajectory, and the standard deviation of the velocity computed over the frame-to-frame instantaneous velocity measurements. The flow profiles shown in Fig. 4 were built by plotting the velocity of the particles as a function of the *y* coordinate of their trajectory.

XYT stacks were also projected along the T coordinate, as explained in the text and in the Supplementary Information (Figs S3 and S4), in order to investigate the structure of the RBC flow within the channels and to estimate the thickness of the cell-depleted layer.

## Additional Information

**How to cite this article:** Tsvirkun, D. *et al*. Microvasculature on a chip: study of the Endothelial Surface Layer and the flow structure of Red Blood Cells. *Sci. Rep.* **7**, 45036; doi: 10.1038/srep45036 (2017).

**Publisher's note:** Springer Nature remains neutral with regard to jurisdictional claims in published maps and institutional affiliations.

## Supplementary Material

Supplementary Information

## References

[b1] Carlos T. & Harlan J. Leukocyte-Endothelial Adhesion Molecules. Blood 84, 2068–2101 (1994). 7522621

[b2] Mackay C. Chemokines: immunology’s high impact factors. Nat. Immunol. 2, 95–101 (2001). 11175800 10.1038/84298

[b3] Wagner D. D. & Frenette P. S. The vessel wall and its interactions. Blood 111, 5271–5281 (2008). 18502843 10.1182/blood-2008-01-078204 PMC2396724

[b4] Langer H. F. & Chavakis T. Leukocyte-endothelial interactions in inflammation. J. Cell. Mol. Med. 13, 1211–1220 (2009). 19538472 10.1111/j.1582-4934.2009.00811.x PMC2861890

[b5] Walsh T. G., Metharom P. & Berndt M. C. The functional role of platelets in the regulation of angiogenesis. Platelets 26, 199–211 (2015). 24832135 10.3109/09537104.2014.909022

[b6] Manegold P., Hutter J., Pahernik S., Messmer K. & Dellian M. Platelet-endothelial interaction in tumor angiogenesis and microcirculation. Blood 101, 1970–1976 (2003). 12584142 10.1182/blood.V101.5.1970

[b7] Hoffman M. & Monroe D. A cell-based model of hemostasis. Thromb. Haemost. 85, 958–965 (2001). 11434702

[b8] Henn V. . CD40 ligand on activated platelets triggers an inflammatory reaction of endothelial cells. Nature 391, 591–594 (1998). 9468137 10.1038/35393

[b9] Gerszten R. . MCP-1 and IL-8 trigger firm adhesion of monocytes to vascular endothelium under flow conditions. Nature 398, 718–723 (1999). 10227295 10.1038/19546

[b10] Hou H. W. . Deformability based cell margination-A simple microfluidic design for malaria-infected erythrocyte separation. Lab Chip 10, 2605–2613 (2010). 20689864 10.1039/c003873c

[b11] Fedosov D. A. & Gompper G. White blood cell margination in microcirculation. Soft Matter 10, 2961–2970 (2014). 24695813 10.1039/c3sm52860j

[b12] Song J. W. & Munn L. L. Fluid forces control endothelial sprouting. Proc. Natl. Acad. Sci. USA 108, 15342–15347 (2011). 21876168 10.1073/pnas.1105316108 PMC3174629

[b13] Kaul D., Fabry M. & Nagel R. Microvascular sites and characteristics of sickle-cell adhesion to vascular endothelium in shear-flow conditions - pathophysiological implications. Proc. Natl. Acad. Sci. USA 86, 3356–3360 (1989). 2497465 10.1073/pnas.86.9.3356 PMC287131

[b14] Rajendran P. . The Vascular Endothelium and Human Diseases. Int. J. Biol. Sci. 9, 1057–1069 (2013). 24250251 10.7150/ijbs.7502 PMC3831119

[b15] Wong K. H. K., Chan J. M., Kamm R. D. & Tien J. Microfluidic Models of Vascular Functions. Annu. Rev. Biomed. Eng. 14, 205–230 (2012). 22540941 10.1146/annurev-bioeng-071811-150052

[b16] Tien J. Microfluidic approaches for engineering vasculature. Curr. Opin. Chem. Eng. 3, 36–41 (2014).

[b17] Chan J. M., Wong K. H. K., Richards A. M. & Drum C. L. Microengineering in cardiovascular research: new developments and translational applications. Cardiovasc. Res. 106, 9–18 (2015). 25691539 10.1093/cvr/cvv049 PMC4362405

[b18] Branchford B. R., Ng C. J., Neeves K. B. & Di Paola J. Microfluidic technology as an emerging clinical tool to evaluate thrombosis and hemostasis. Thromb. Res. 136, 13–19 (2015). 26014643 10.1016/j.thromres.2015.05.012 PMC4910695

[b19] Skommer J. & Wlodkowic D. Successes and future outlook for microfluidics-based cardiovascular drug discovery. Expert. Opin. Drug. Discov. 10, 231–244 (2015). 25672221 10.1517/17460441.2015.1001736

[b20] Shin M. . Endothelialized networks with a vascular geometry in microfabricated poly(dimethyl siloxane). Biomed. Microdevices 6, 269–278 (2004). 15548874 10.1023/B:BMMD.0000048559.29932.27

[b21] Chrobak K., Potter D. & Tien J. Formation of perfused, functional microvascular tubes *in vitro*. Microvasc. Res. 71, 185–196 (2006). 16600313 10.1016/j.mvr.2006.02.005

[b22] Rosano J. M. . A physiologically realistic *in vitro* model of microvascular networks. Biomed. Microdevices 11, 1051–1057 (2009). 19452279 10.1007/s10544-009-9322-8 PMC2888702

[b23] Fiddes L. K. . A circular cross-section PDMS microfluidics system for replication of cardiovascular flow conditions. Biomaterials 31, 3459–3464 (2010). 20167361 10.1016/j.biomaterials.2010.01.082

[b24] Kim S., Lee H., Chung M. & Jeon N. L. Engineering of functional, perfusable 3D microvascular networks on a chip. Lab Chip 13, 1489–1500 (2013). 23440068 10.1039/c3lc41320a

[b25] Golden A. P. & Tien J. Fabrication of microfluidic hydrogels using molded gelatin as a sacrificial element. Lab Chip 7, 720–725 (2007). 17538713 10.1039/b618409j

[b26] Bertassoni L. E. . Hydrogel bioprinted microchannel networks for vascularization of tissue engineering constructs. Lab Chip 14, 2202–2211 (2014). 24860845 10.1039/c4lc00030g PMC4201051

[b27] Tsai M. . *In vitro* modeling of the microvascular occlusion and thrombosis that occur in hematologic diseases using microfluidic technology. J. Clin. Invest. 122, 408–418 (2012). 22156199 10.1172/JCI58753 PMC3248292

[b28] Morgan J. P. . Formation of microvascular networks *in vitro*. Nat. Protoc. 8, 1820–1836 (2013). 23989676 10.1038/nprot.2013.110

[b29] Zheng Y. . *In vitro* microvessels for the study of angiogenesis and thrombosis. Proc. Natl. Acad. Sci. USA 109, 9342–9347 (2012). 22645376 10.1073/pnas.1201240109 PMC3386137

[b30] Mannino R. G. . Do-it-yourself *in vitro* vasculature that recapitulates *in vivo* geometries for investigating endothelial-blood cell interactions. Sci. Rep. 5 (2015). 10.1038/srep12401 PMC4894411 26202603

[b31] Myers D. R. . Endothelialized Microfluidics for Studying Microvascular Interactions in Hematologic Diseases. J. Vis. Exp.(2012). 10.3791/3958 PMC3471282 22760254

[b32] Rollins M. R., Ahn B., Sakurai Y. & Lam W. A. Characterizing Cellular Interactions Contributing to Vaso-Occlusion in Patients with Sickle Cell Disease Utilizing a Novel Endothelialized Microfluidic Device. Blood 126 (2015).

[b33] Ciciliano J. C. . Resolving the multifaceted mechanisms of the ferric chloride thrombosis model using an interdisciplinary microfluidic approach. Blood 126, 817–824 (2015). 25931587 10.1182/blood-2015-02-628594 PMC4528067

[b34] Zheng Y., Chen J. & Lopez J. A. Microvascular platforms for the study of platelet-vessel wall interactions. Thromb. Res. 133, 525–531 (2014). 24438943 10.1016/j.thromres.2013.12.039 PMC4692378

[b35] Lamberti G. . Bioinspired Microfluidic Assay for *In Vitro* Modeling of Leukocyte Endothelium Interactions. Anal. Chem. 86, 8344–8351 (2014). 25135319 10.1021/ac5018716 PMC4139165

[b36] Fay M. E. . Cellular softening mediates leukocyte demargination and trafficking, thereby increasing clinical blood counts. Proc. Natl. Acad. Sci. USA 113, 1987–1992 (2016). 26858400 10.1073/pnas.1508920113 PMC4776450

[b37] Weinbaum S., Tarbell J. M. & Damiano E. R. The structure and function of the endothelial glycocalyx layer. Annu. Rev. Biomed. Eng. 9, 121–167 (2007). 17373886 10.1146/annurev.bioeng.9.060906.151959

[b38] Pries A., Secomb T. & Gaehtgens P. The endothelial surface layer. Pflug. Arch. Eur. J. Phy. 440, 653–666 (2000). 10.1007/s004240000307 11007304

[b39] Curry F. E. & Adamson R. H. Endothelial Glycocalyx: Permeability Barrier and Mechanosensor. Ann. Biomed. Eng. 40, 828–839 (2012). 22009311 10.1007/s10439-011-0429-8 PMC5042904

[b40] Marki A., Esko J. D., Pries A. R. & Ley K. Role of the endothelial surface layer in neutrophil recruitment. J. Leukoc. Biol. 98, 503–515 (2015). 25979432 10.1189/jlb.3MR0115-011R PMC4569049

[b41] Tarbell J. M., Simon S. I. & Curry F.-R. E. Mechanosensing at the Vascular Interface. Annu. Rev. Biomed. Eng. 16, 505–532 (2014). 24905872 10.1146/annurev-bioeng-071813-104908 PMC4450720

[b42] Pries A. & Secomb T. Microvascular blood viscosity *in vivo* and the endothelial surface layer. Am. J. Physiol. Heart Circ. Physiol. 289, H2657–H2664 (2005). 16040719 10.1152/ajpheart.00297.2005

[b43] Damiano E. The effect of the endothelial-cell glycocalyx on the motion of red blood cells through capillaries. Microvasc. Res. 55, 77–91 (1998). 9473411 10.1006/mvre.1997.2052

[b44] Reitsma S., Slaaf D. W., Vink H., van Zandvoort M. A. M. J. & Egbrink M. G. A. o. The endothelial glycocalyx: composition, functions, and visualization. Pflug. Arch. Eur. J. Phy. 454, 345–359 (2007). 10.1007/s00424-007-0212-8 PMC1915585 17256154

[b45] Salmon A. H. J. & Satchell S. C. Endothelial glycocalyx dysfunction in disease: albuminuria and increased microvascular permeability. J. Pathol. 226, 562–574 (2012). 22102407 10.1002/path.3964

[b46] Potter D. R. & Damiano E. R. The hydrodynamically relevant endothelial cell glycocalyx observed *in vivo* is absent *in vitro*. Circ. Res. 102, 770–776 (2008). 18258858 10.1161/CIRCRESAHA.107.160226

[b47] Chappell D. . The Glycocalyx of the Human Umbilical Vein Endothelial Cell An Impressive Structure *Ex Vivo* but Not in Culture. Circ. Res. 104, 1313–1317 (2009). 19423849 10.1161/CIRCRESAHA.108.187831

[b48] Ebong E. E., Macaluso F. P., Spray D. C. & Tarbell J. M. Imaging the Endothelial Glycocalyx *In Vitro* by Rapid Freezing/Freeze Substitution Transmission Electron Microscopy. Arterioscler. Thromb. Vasc. Biol. 31, 1908–1915 (2011). 21474821 10.1161/ATVBAHA.111.225268 PMC3141106

[b49] Barker A. . Observation and characterisation of the glycocalyx of viable human endothelial cells using confocal laser scanning microscopy. Phys. Chem. Chem. Phys. 6, 1006–1011 (2004).

[b50] Zeng Y. & Tarbell J. M. The Adaptive Remodeling of Endothelial Glycocalyx in Response to Fluid Shear Stress. PLoS One 9 (2014). 10.1371/journal.pone.0086249 PMC3896483 24465988

[b51] Alroy J., Goyal V. & Skutelsky E. Lectin Histochemistry Of Mammalian Endothelium. Histochemistry 86, 603–607 (1987). 3610672 10.1007/BF00489554

[b52] Kataoka H. . Fluorescent Imaging of Endothelial Glycocalyx Layer With Wheat Germ Agglutinin Using Intravital Microscopy. Microsc. Res. Tech. 79, 31–37 (2016). 26768789 10.1002/jemt.22602

[b53] Salmon A. H. J. . Loss of the Endothelial Glycocalyx Links Albuminuria and Vascular Dysfunction. J. Am. Soc. Nephrol. 23, 1339–1350 (2012). 22797190 10.1681/ASN.2012010017 PMC3402289

[b54] Vargas-Pinto R., Gong H., Vahabikashi A. & Johnson M. The Effect of the Endothelial Cell Cortex on Atomic Force Microscopy Measurements. Biophys. J. 105, 300–309 (2013). 23870251 10.1016/j.bpj.2013.05.034 PMC3714930

[b55] Bugliarello G. & Hayden J. W. Detailed Characteristics of the Flow of Blood *in Vitro*. Trans. Soc. Rheol. 7, 209–230 (1963).

[b56] Wang H. & Wang Y. Flow in microchannels with rough walls: flow pattern and pressure drop. J Micromech Microeng. 17, 586–596 (2007).

[b57] Tuck E. & Kouzoubov A. A laminar roughness boundary-condition. J. Fluid Mech. 300, 59–70 (1995).

[b58] Yen W.-Y., Cai B., Zeng M., Tarbell J. M. & Fu B. M. Quantification of the endothelial surface glycocalyx on rat and mouse blood vessels. Microvasc. Res. 83, 337–346 (2012). 22349291 10.1016/j.mvr.2012.02.005 PMC3371771

[b59] Vink H. & Duling B. Identification of distinct luminal domains for macromolecules, erythrocytes, and leukocytes within mammalian capillaries. Circ. Res. 79, 581–589 (1996). 8781491 10.1161/01.res.79.3.581

[b60] Devaraj S., Yun J.-M., Adamson G., Galvez J. & Jialal I. C-reactive protein impairs the endothelial glycocalyx resulting in endothelial dysfunction. Cardiovasc. Res. 84, 479–484 (2009). 19620133 10.1093/cvr/cvp249 PMC2777951

[b61] Pries A. . Microvascular blood flow resistance: role of endothelial surface layer. Am. J. Physiol. Heart Circ. Physiol. 273, H2272–H2279 (1997). 10.1152/ajpheart.1997.273.5.H2272 9374763

[b62] Lanotte L., Tomaiuolo G., Misbah C., Bureau L. & Guido S. Red blood cell dynamics in polymer brush-coated microcapillaries: A model of endothelial glycocalyx *in vitro*. Biomicrofluidics 8, 014104 (2014). 24753725 10.1063/1.4863723 PMC3977877

[b63] Tateishi N., Suzuki Y., Soutani M. & Maeda N. Flow dynamics of erythrocytes in microvessels of isolated rabbit mesentery - cell-free layer and flow resistance. J. Biomech. 27, 1119–1125 (1994). 7929461 10.1016/0021-9290(94)90052-3

[b64] Kim S., Kong R. L., Popel A. S., Intaglietta M. & Johnson P. C. Temporal and spatial variations of cell-free layer width in arterioles. Am. J. Physiol. Heart Circ. Physiol. 293, H1526–H1535 (2007). 17526647 10.1152/ajpheart.01090.2006

[b65] Kim S., Ong P. K., Yalcin O., Intaglietta M. & Johnson P. C. The cell-free layer in microvascular blood flow. Biorheology 46, 181–189 (2009). 19581726 10.3233/BIR-2009-0530

[b66] Dietzel S. . Label-Free Determination of Hemodynamic Parameters in the microcirculaton with Third Harmonic Generation Microscopy. PLoS One 9, e99615 (2014). 24933027 10.1371/journal.pone.0099615 PMC4059650

[b67] Suzuki Y., Tateishi N., Soutani M. & Maeda N. Flow behavior of erythrocytes in microvessels and glass capillaries: Effects of erythrocyte deformation and erythrocyte aggregation. Int. J. Microcirc. 16, 187–194 (1996). 10.1159/000179172 8923151

[b68] Grandchamp X., Coupier G., Srivastav A., Minetti C. & Podgorski T. Lift and Down-Gradient Shear-Induced Diffusion in Red Blood Cell Suspensions. Phys. Rev. Lett. 110, 108101 (2013). 23521300 10.1103/PhysRevLett.110.108101

[b69] Zhao H., Shaqfeh E. S. G. & Narsimhan V. Shear-induced particle migration and margination in a cellular suspension. Phys. Fluids 24, 011902 (2012).

[b70] Fedosov D. A., Caswell B. & Popel A. S. & Karniadakis, G. E. Blood Flow and Cell-Free Layer in Microvessels. Microcirculation 17, 615–628 (2010). 21044216 10.1111/j.1549-8719.2010.00056.x PMC3529161

[b71] Shen Z. . Inversion of hematocrit partition at microfluidic bifurcations. Microvasc. Res. 105, 40–46 (2016). 26744089 10.1016/j.mvr.2015.12.009

[b72] Rival Y., DelMaschio A., Rabiet M., Dejana E. & Duperray A. Inhibition of platelet endothelial cell adhesion molecule-1 synthesis and leukocyte transmigration in endothelial cells by the combined action of TNF-alpha and IFN-gamma. J. Immunol. 157, 1233–1241 (1996). 8757631

[b73] Schindelin J. . Fiji: an open-source platform for biological-image analysis. Nat. Methods 9, 676–682 (2012). 22743772 10.1038/nmeth.2019 PMC3855844

