# Supplementary material for: Microvasculature on a chip: study of the Endothelial Surface Layer and the flow structure of Red Blood Cells
Source: Sci Rep. 2017 Mar 24;7:45036. doi: 10.1038/srep45036 (PMC5364477; doi:10.1038/srep45036)
Supplement: Supplementary Information [file srep45036-s1.pdf]

Supplementary Information

Microvasculature on a chip: study of the Endothelial Surface Layer and the flow structure of Red Blood Cells

Daria Tsvirkun[1,2,5], Alexei Grichine[3,4], Alain Duperray[3,4], Chaouqi Misbah[1,2], and Lionel Bureau[1,2,*]

[1]Univ. Grenoble Alpes, LIPHY, F-38000 Grenoble, France.
[2]CNRS, LIPHY, F-38000 Grenoble, France.
[3]Univ. Grenoble Alpes, IAB, F-38000 Grenoble, France.
[4]INSERM, IAB, F-38000 Grenoble, France.
[5]Research Center for Obstetrics, Gynecology and Perinatology. 4, Oparin street, Moscow, Russian Federation 117997.
[*]lionel.bureau@univ-grenoble-alpes.fr

Neuraminidase treatment

In order to assess the binding of WGA to the endothelial surface layer of the cells, we have performed the following control experiments with HUVEC plated in standard culture wells. Cultured cells stained with WGA-Alexa have been exposed for 60 minutes at 37°C to neuraminidase (1 U/mL in culture medium), and XYZ image stacks have been taken before and after such an enzymatic treatment, known to digest the glycocalyx of the HUVEC[1].

As illustrated in Fig. S1A and B, we observe, on XY images taken at the same Z with respect to the bottom surface, that exposure to neuraminidase induces a decrease, by 40-50% (Fig. S1E), in the detected fluorescence intensity. This indicates, in agreement with previous works[1], that WGA indeed binds to the glycocalyx, and that the latter has been partially removed upon exposure to neuraminidase. This is further confirmed by comparing XZ cross-sections of the stacks (see Fig. S1C and D), which show that after enzymatic digestion, cells display a surface layer that is much less homogeneous and much dimmer than before treatment.

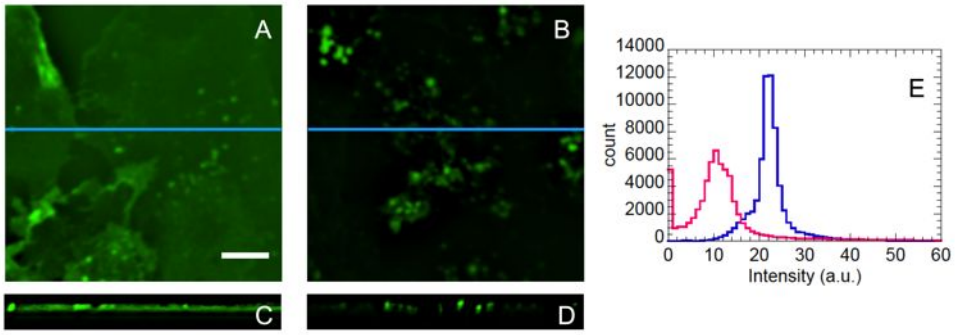

Figure S1. XY images of WGA-Alexa stained HUVEC before (A) and after (B) neuraminidase treatment (Scale bar: 10 μm). XZ images (along blue line on A and B) before (C) and after (D) enzymatic digestion, showing less bright and more heterogeneous ESL after neuraminidase treatment. (E) Intensity histograms of images A and B, showing a shift in average fluorescence intensity from 22 (blue histogram) to 12 (purple histogram) upon ESL digestion.

Peripheral cell body thickness

The average peripheral thickness of the cellular bodies, h_{peri}, which sets the minimum distance between the bare channel wall and the apical membrane of the cells, have been measured as illustrated in Fig. S2: XY CFM images of HUVEC adhered on the lateral walls of the channels and whose cytoplasm had been stained (Fig. S2A) were used to measure h_{peri} from intensity profiles (Fig. S2B) plotted across the cell body far away from the nucleus. Doing so over 65 measurement points at different locations in the channels, we have obtained a value of $h_{peri} = 0.9 \pm 0.2\,\mu$m (Fig. S2C).

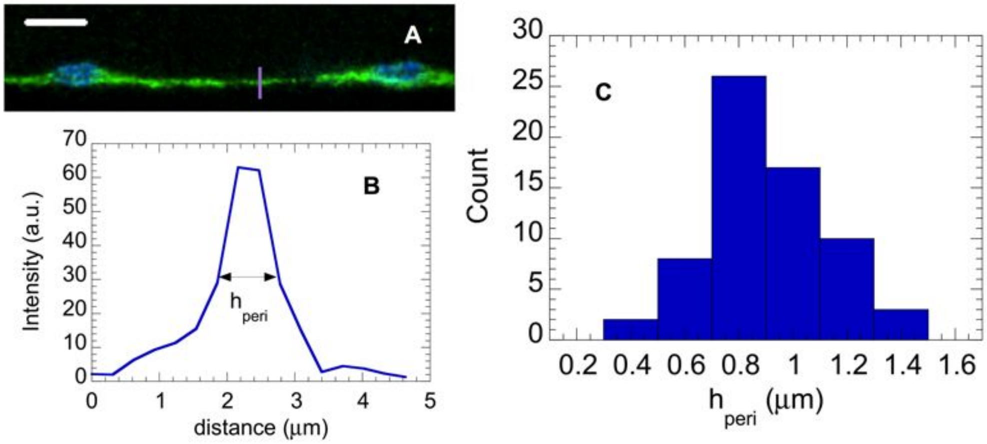

Figure S2. (A) CFM image of two adjacent HUVEC adhered on a lateral wall (cytoplasm in green, nuclei in blue). Scale bar: 10 μm. (B) Intensity profile along the purple line drawn in (A). h_{peri} is measured as the full width at mid height of the intensity peak. (C) Histogram of the measured peri-cellular thickness h_{peri}.

Image processing

Fig. S3 presents the various image processing steps of the particle tracking velocimetry scheme used in our study. The initial XYT image stack (S3A) is first projected along the T coordinate to compute a time-averaged intensity image (AVG image, Fig. S3B) of the stack, in which the intensity of each pixel corresponds to its intensity averaged over the number of frame in the stack. This leaves apparent only the stained ESL at the channel walls, which is bright on all the frames, while the intensity of pixels away from the ESL, which are bright only for a limited number of frames when a red blood cell is crossing, is on average close to the dark background value. The AVG image is then subtracted to all the frames of the initial stack, yielding a "wall-subtracted" stack in which only the flowing RBC are visible (Fig. S3C). This is done so as to avoid any disturbance caused by the intensity from the ESL in the detection of RBC flowing near the walls. On such a stack, RBC are seen to display, due to their discocyte shape, a maximum intensity at their periphery and a minimum at their center. In order for the automated particle detection plugin to track accurately the location of the RBC center of mass, we apply to the wall-subtracted stack a gaussian blur filter (Fig. S3D). We choose a kernel of 3 pixels in radius, which allows us to make the location of the intensity maximum coincide with the center of mass of the RBC, as illustrated on Fig. S3E. Following this, cell tracking, trajectory building (Fig. S3F) and velocimetry are performed using the TrackMate plugin of Fiji, as described in the Methods section of the manuscript.

Image stacks are further processed as follows in order to investigate the spatial distribution of RBC across the channel width. First, a second order derivative of the AVG image is performed to produce an image of the ESL with enhanced contrast (Fig. S3H). Such an image is used to determine the local width of the channel, from ESL to ESL, by measuring the distance separating the inner mid-height points of the two lateral peaks displayed by intensity profiles such as that shown in Fig. S3I (we have checked that doing the same directly on the unprocessed AVG image yielded a value of the channel width within about 0.2-0.3 μm of that determined from the 2nd-derivative image). Second, the gaussian-blurred image stack of the flowing RBC is projected along the T coordinate to compute the standard deviation of pixel intensity over the number of frames (STD-DEV image, Fig. S3G). In contrast to the AVG projection, a STD-DEV projection highlights the regions where intensity fluctuations are large. These regions correspond, in our stacks, to those where intensity fluctuates in time due to the traveling RBC. Such a STD-DEV projection along the time coordinate therefore produces an image containing the overlay of the paths taken by the RBC over the duration of the stack, and thus provides the time-averaged picture of the flowing RBC column. From such an image, we estimate the width of the flowing column, which we take as the width at mid-height of the broad and structured intensity bump displayed by intensity profiles plotted across the STD-DEV image (see Fig. S3I). The channel and column

width measurements are then used to compute the thickness of the near-wall cell-depleted layer, as described in the manuscript.

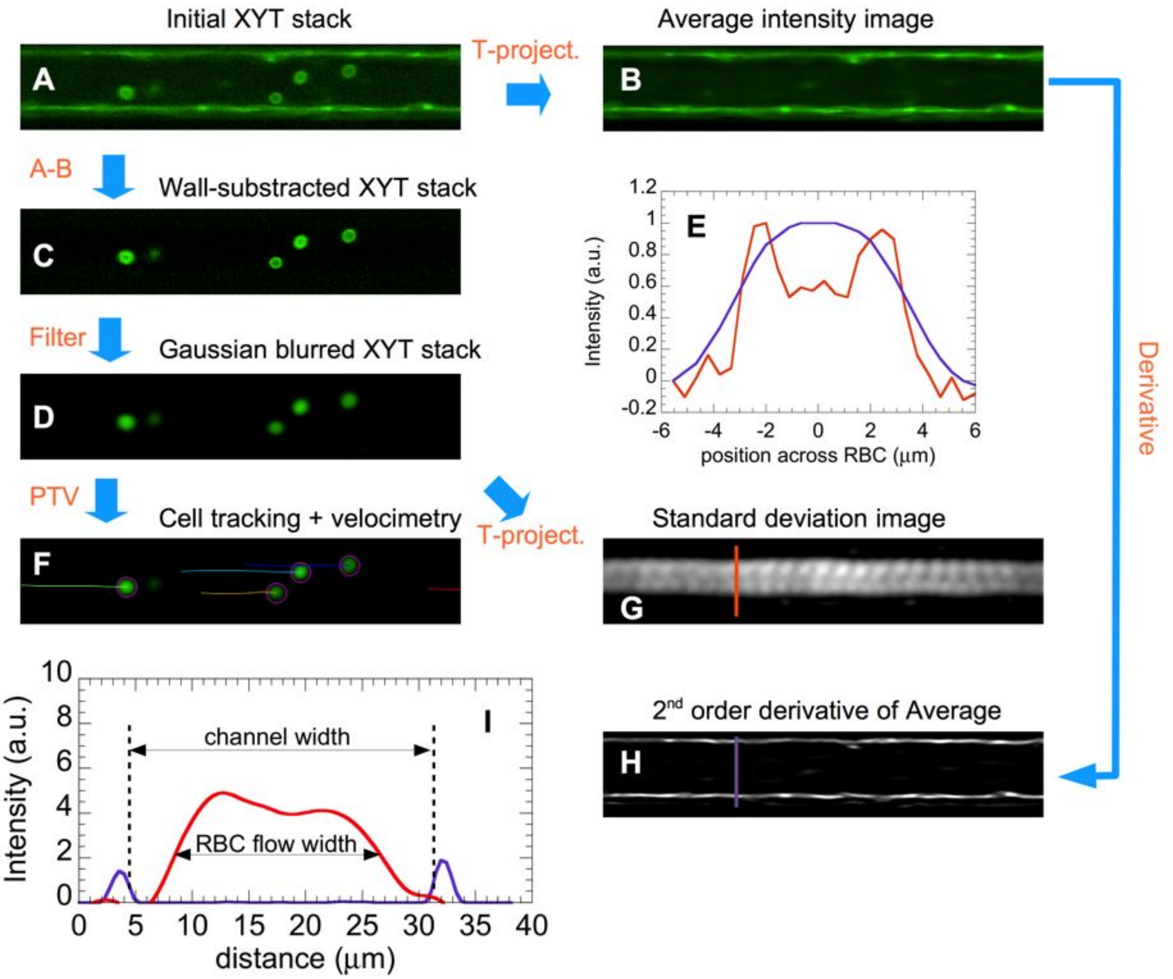

Figure S3. Flowchart for image processing and analysis. (A) Initial XYT image stack showing the ESL at the wall and the RBC. (B) Average intensity T-projection image of (A). (C) Result of the subtraction of (A) and (B), leaving only the RBC visible. (D) Result of the gaussian Blur filter applied to (C). (E) Intensity profiles across a RBC before (orange line) and after (purple line) gaussian filtering. (F) Example of RBC tracking: detected RBC are circled in purple, and their trajectory over the six subsequent images are shown as overlaid colored lines (flow is from right to left). (G) Standard deviation T-projection of XYT stack (D), revealing the paths of RBC. (H) Result of a second-order derivative of image (B), highlighting the position of the ESL. (I) Intensity profiles plotted along the lines drawn in (G, orange line) and (H, purple line), from which the width of the channel and the RBC flowing column are determined. All images are $227.5 \times 44.5\,\mu$m in size.

As shown in Fig. S3E, the gaussian filter applied during the analysis process smoothes out the sharp features of the RBC. This, along with the fact that we rely on STD-DEV projections to quantify the width of the RBC column, may affect our estimate of the thickness of the cell-depleted layer. We have therefore compared the value of the width obtained as above with that determined from a projection of the maximum intensity performed on unfiltered wall-subtracted stacks (MAX projection, where each pixel is given its maximum intensity over the stack duration). Similarly to what we describe in the manuscript, an overlay of the MAX and AVG projections of the stack again reveals a dark near-wall layer (see Fig. S4A) from which we can unambiguously conclude to the existence of a region close to the ESL that is depleted in RBC. A comparison of the two projection images (MAX and STD-DEV) is provided in Fig. S4B and C. It can be seen on this figure that the MAX projection performed on the non-filtered stack yields an image in which the periphery of the flowing RBC is clearly seen, including

near the walls. Intensity profiles plotted across such an image display several peaks, among which the two outermost ones indicate the edges of the RBC traveling closest to the channel walls (Fig. S4D). Taking the distance between these two peaks as a measure of the column width, we typically obtain values that are only about 0.5-0.7 μm larger than what we determine from the smoothed profiles, as illustrated in Fig. S4D and E. Overall, the bias introduced by image processing/filtering in the determination of channel and column width is therefore below 1 μm, *i.e.* less than 0.5 μm on the estimate of the thickness of the cell-depleted layer, which is comparable with the spreading of the data from point-to-point measurement, as mentioned in the manuscript.

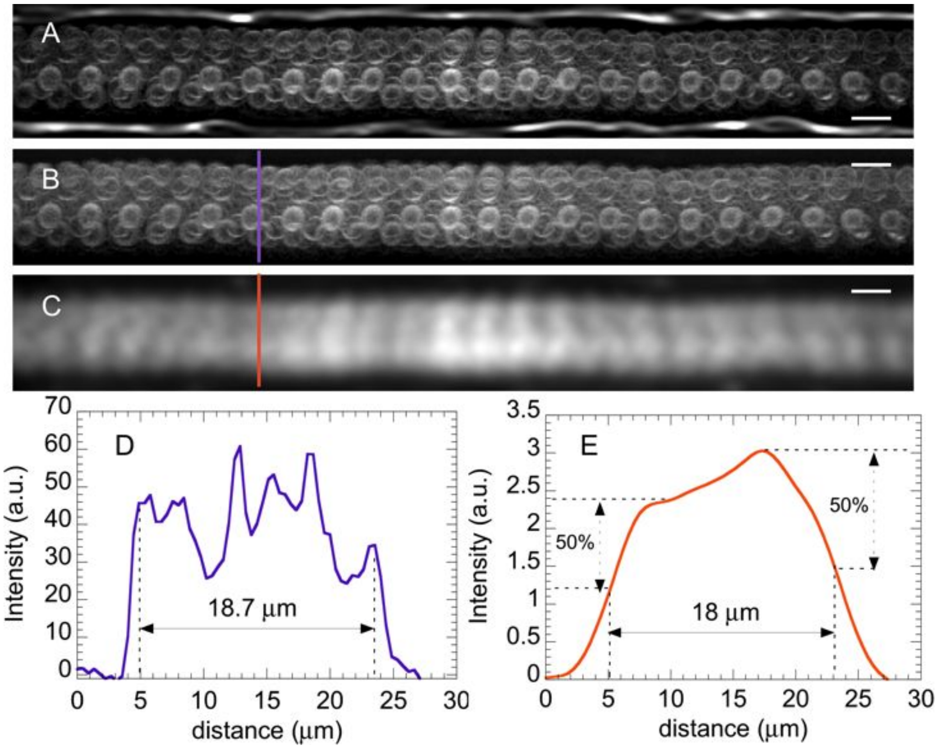

Figure S4. (A) Overlay of MAX and AVG projections, showing the presence of a near-wall layer where image intensity is zero. (B) MAX projection of a non-filtered wall-subtracted stack. (C) STD-DEV projection of the same stack after application of gaussian filter. Scale bar on (A)-(C): 10 μm. (D) Intensity plot along the line drawn on (B), leading to a RBC column width of 18.7 μm. (E) Intensity plot along the line drawn on (C), leading to a RBC column width of 18 μm.

Channel dimensions and flow profiles in bare channels

The actual dimensions of the fabricated microchannels are determined by cutting, with a scalpel, thin sections of the circuit across the channels of interest. Sections are then laid flat on a glass coverslip, and imaged by optical microscopy in reflection, using a 20x NA 0.75 air objective and a high resolution camera yielding a sampling of 0.185 μm per pixel. An example of the obtained images is provided in Fig. S5A. Channel width is taken as the peak-to-peak distance of intensity profiles plotted across the channel (Fig. S5A and C). Channel height is determined using a similar procedure with vertical intensity profiles plotted across the channel ceiling and a line drawn manually and prolonging the position of the bottom PDMS surface visible on each side of the channel (Fig. S5B). Width and height are respectively measured at two different positions in the channel and over the 16 microchannels of interest. The histogram presented in Fig. S5D shows that this procedure, performed on channels of $30 \times 30\,\mu\text{m}^2$ nominal cross-section, yields an actual channel size of $32 \pm 0.5\,\mu$m.

Building upon the above procedure for the determination of actual channel dimensions, we have checked the agreement between the flow velocity profiles measured experimentally in bare microchannels and the expected Poiseuille profiles. The latter are computed as $V(y) = V_{max}[1-(y/y_{wall})^2]$, where, as explained in the manuscript, $V_{max} = 2Q/(16S)$ in each of the 16 parallel channels, with Q the imposed flow rate, S the actual channel cross-section, and y_{wall} the wall position with respect to the central axis. A comparison of the measured and expected profiles is illustrated in Fig. S6. It can be seen that, within the uncertainty on actual channel dimensions, the quantitative agreement is excellent.

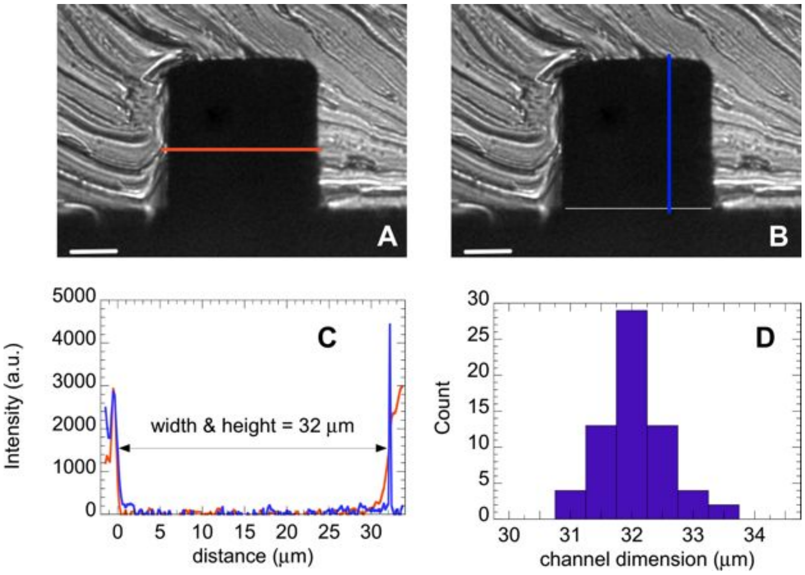

Figure S5. (A) optical image in reflection of a PDMS thin slice, showing the cross section (appearing in black) of a channel of nominal dimension $30 \times 30\,\mu$m (scale bar is 10 μm). (B) same image with a thin horizontal line drawn manually and aligned with the bottom part of the PDMS slab. (C) intensity profiles along the orange and blue lines drawn in A and B, from which the actual width and height of the channel, here equal to 32 μm, are determined. (D) Histogram of measured channel dimensions.

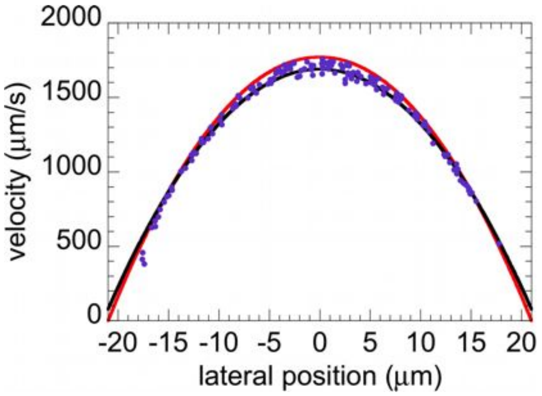

Figure S6. Flow velocity profile (symbols) measured at mid-height of a channel having an actual lateral dimension of $42.5 \pm 0.5\,\mu$m, perfused at an imposed global flow rate of 1.5 μL.min^{-1}. The channel to channel reproducibility of such a profile was found to be of $\pm 130\,\mu$m.s^{-1} over the 16 central channels of the circuit. For comparison, the theoretical profiles expected from Poiseuille theory are plotted for a channel lateral dimension of 42 μm (red line) and 43 μm (black line).

References

1. Barker, A. *et al.* Observation and characterisation of the glycocalyx of viable human endothelial cells using confocal laser scanning microscopy. *Phys. Chem. Chem. Phys.* **6**, 1006–1011 (2004).
